# Association between Excessive Use of Mobile Phone and Insomnia and Depression among Japanese Adolescents

**DOI:** 10.3390/ijerph14070701

**Published:** 2017-06-29

**Authors:** Haruka Tamura, Tomoko Nishida, Akiyo Tsuji, Hisataka Sakakibara

**Affiliations:** 1 Department of Nursing, Nagoya University Graduate School of Medicine, 1-1-20 Daiko-Minami, Higashi-ku, Nagoya 461-8673, Japan; tsuji.akiyo@f.mbox.nagoya-u.ac.jp (A.T.); sbara@met.nagoya-u.ac.jp (H.S.); 2 Department of Nursing, Sugiyama Jogakuen University, 17-3 Hoshigaoka-Motomachi, Chikusa-Ku, Nagoya 464-8662, Japan; t-nishida@sugiyama-u.ac.jp

**Keywords:** adolescents, depression, insomnia, mobile phone, Japanese

## Abstract

The aim of this study was to investigate the relationship between mobile phone use and insomnia and depression in adolescents. A cross-sectional study was conducted on 295 high school students aged 15–19 in Japan. Insomnia and depression were assessed using Athene Insomnia Scales (AIS) and the Center for Epidemiologic Studies Depression Scale (CES-D), respectively. Mobile phones were owned by 98.6% of students; 58.6% used mobile phones for over 2 h per day and 10.5% used them for over 5 h per day. Overall mobile phone use of over 5 h per day was associated with shorter sleep duration and insomnia (OR: 3.89 [95% CI: 1.21–12.49]), but not with depression. Mobile phone use of 2 h or more per day for social network services (OR: 3.63 [1.20–10.98]) and online chats (OR: 3.14 [1.42–6.95]), respectively, was associated with a higher risk of depression. Mobile phone overuse can be linked to unhealthy sleep habits and insomnia. Moreover, mobile phone overuse for social network services and online chats may contribute more to depression than the use for internet searching, playing games or viewing videos.

## 1. Introduction

The distribution of mobile phones in Japanese adolescents has risen rapidly. According to the Cabinet Office, Government of Japan, the distribution rate of mobile phones in adolescents aged 10–17 was 52.6% in 2011, 59.5% in 2013, and 68.3% in 2015 [1]. For instance, in 2015, 96.7% of senior high school students had mobile phones [1]. Many mobile phone users now have the most advanced version, called a smartphone. A smartphone is a useful tool that enables access to the internet and social networks, messaging, viewing videos, and playing games. Therefore, comparatively more hours are spent on a smartphone than on a conventional phone [2]. In 2008, less than 40% of adolescents used mobile phones for more than 2 h per day [3], while in 2015 about 50% of adolescents used smartphones for more than 3 h per day [4]. In Japan, smartphones have also come to be used widely. Approximate 95% of Japanese senior high school students use them [1]. Among Asian countries, Japanese adolescents in particular engage in various internet applications such as online gaming, blogging, instant messenger and e-mail [5]. It is predicted that smartphone use may increase drastically in the future.

The mobile phone is reportedly a useful tool for health promotion. Mobile applications offer effective ways to improve one’s lifestyle, for example, by increasing physical activity [6], weight control [7,8], and treating obesity [9]. On the other hand, mobile phone use could cause physical [10,11,12] and psychological [3,13,14] health problems when used excessively. It is reported that mobile phone use in bed at night negatively impacts sleep outcome [3,15,16]. This may be due to exposure to bright light from electronic devices, disturbing the circadian rhythms and then sleep quality [16,17,18]. Mobile phone overuse may also pose the risk of mobile phone addiction or smartphone addiction [4,14], thus contributing to poor sleep quality [13,14,19,20] and psychological problems such as depression and anxiety [13,21,22]. Similar risks associated with mobile phone overuse are also reported among Japanese adolescents [23,24].

Nowadays, adolescents are likely to use mobile phones for more hours per day, which can lead to poor sleep and psychological problems. However, there are only few studies examining the relationship between hours of mobile phone use per day and health problems among adolescents. Focusing on overall hours of mobile phone use per day and hours spent per purpose of use, the aim of this study was to investigate the associations between mobile phone overuse and insomnia and depression in senior Japanese high school students.

## 2. Materials and Methods

### 2.1. Design and Sample

A cross-sectional study was conducted using self-reported questionnaires. Participants were recruited from one public senior high school in Gifu Prefecture, Japan, between June and July 2014. This school is a prefectural high school in a local city, Japan, which has several courses such as a general course, an agriculture course, an animal husbandry course, a business course, and an information processing course. About 40% of students go on to university or technical college, and others get a job. This school comprised 346 students (120 in the 10th grade, 117 in the 11th grade, and 109 in the 12th grade). The first to third grades of high school in Japan correspond to the 10th to 12th grades in the U.S. Anonymous questionnaires were distributed to these 346 students after their class teacher had explained the nature of the study. Students returned the questionnaires in a sealed envelope to ensure confidentiality of their information. Of the 346 students, 332 (96.0%) agreed to participate in this study. After excluding 37 questionnaires with incomplete information on mobile phone use, insomnia or depression, 295 (88.9%) were analyzed. This study was approved by the research and ethics committee of the School of Medicine at the Graduate School of Nagoya University (14-102).

### 2.2. Measurements

The self-administered questionnaires included question items on (1) personal data; (2) lifestyle; (3) social support; (4) mobile phone use; (5) insomnia; and (6) depression.

#### 2.2.1. Personal Data

Personal data were gathered about participants’ sex, age, and school grade.

#### 2.2.2. Lifestyle

Question items related to lifestyle included participation in school club activities, eating breakfast, having a talk with family, wake-up time, bedtime, and sleeping hours. Participants were asked to select responses regarding participation in school club activities (sports club, culture club, and none). Similarly, they answered a question on eating breakfast (eat daily and occasionally do not eat) and talking with family (talk daily or occasionally do not talk). Participants were also questioned on wake-up time (earlier than 06:00, at 06:00–07:00, and later than 07:00), bedtime (earlier than 23:00, at 23:00–00:00, at 00:00–01:00, and later than 01:00), and sleeping hours (<5 h, 5 to 6 h, 6 to 7 h, and ≥7 h).

#### 2.2.3. Mobile Phone Use

Mobile phone use was determined by the question “Do you own a mobile phone?” (own a smartphone, own a conventional phone, and none). Hours of mobile phone use per day was assessed by the following question: “How many hours per day do you usually spend on a mobile phone during a typical day?” The responses were: none, <1 h, 1 to 2 h, 2 to 3 h, 3 to 4 h, 4 to 5 h, ≥5 h. The responses regarding purposes of use, such as for e-mails, social networking sites (SNS) (e.g., Facebook, Twitter, Instagram), online chat (e.g., Line, Skype, Kakao Talk), internet searching, playing games, and viewing videos were: none, <30 min, 30 to 60 min, 60 to 120 min, ≥120 min.

#### 2.2.4. Depression

Depression was evaluated using the Japanese version of the Center for Epidemiological Studies-Depression (CES-D) scale, which is a 20-item self-administered questionnaire [25]. CES-D, developed by Radloff [26], is widely used in many countries to assess depressive symptoms of general populations including adolescents [26,27]. Its reliability and validity have been demonstrated [25,26]. Internal consistency as measured by Cronbach’s alpha is reported to be around 0.85 in community samples [26] and adolescents [27,28]. Cronbach’s alpha in the current study was 0.83. This scale assesses the frequency of depressive symptoms experienced during the last week (0: rarely or never; 1: sometimes or on rare occasions; 2: occasionally or a moderate amount of time; and 3: most or all of the time). The scores range from 0 to 60, where a higher score indicates more severe depression. A score of 16 or greater is used to define clinically meaningful depressive symptoms [26,27]. Hence, in this study, the cut-off value of 16 points was used to identify students with depression.

#### 2.2.5. Insomnia

Insomnia was evaluated using the Japanese version of the Athens Insomnia Scale (AIS) [29]. This scale is widely used as a useful tool to assess insomnia [29,30]. Its reliability and validity have been demonstrated. Cronbach’s alpha values are 0.88 in community samples [29] and around 0.81 in adolescents [31,32]. Cronbach’s alpha was 0.79 in this study. This scale consists of eight items: difficulty with sleep induction; waking up during the night; waking up early in the morning ; total sleep time; overall quality of sleep; problems with sense of well-being; problems with functioning; and sleepiness during the day. Each item is rated on a scale of 0 (no problem) to 3 (serious problem), and the range of the total score is from 0 to 24. An AIS score of six points is the optimum cutoff based on the ICD-10 diagnosis of insomnia [30]. Therefore, also in this study, the cut-off value of six points was used to identify insomnia.

#### 2.2.6. Social Support

Social support was evaluated using the Japanese brief version of the Multidimensional Scale of Perceived Social Support (MSPSS), which is a 7-item self-administered questionnaire [33,34]. This scale measures perceived social support from family, friends, and a significant other. Its reliability and validity have been shown. Cronbach’s alpha values for MSPSS are reportedly 0.85 in community samples [34], and 0.83 and 0.93 in adolescents [35,36]. Cronbach’s alpha was 0.93 in this study. Each item is rated on a 7-point Likert scale, and the total score is calculated by averaging the scores for all items. The scores range from 1 to 7, and a higher score indicates better social support.

### 2.3. Statistical Analysis

Differences between mobile phone use and insomnia, depression, and the other health constructs were statistically tested using the Mantel–Haenszel test for trend and the Kruskal–Wallis test. Associations between mobile phone use and insomnia or depression were examined using multiple logistic regression analyses. The dependent variable was insomnia (0 = no problem [AIS score <6] and 1 = insomnia [AIS score ≥6]) or depression (0 = no problem [CES-D score <6] and 1 = depression [CES-D score ≥16)]). Odds ratio (OR) was calculated from the logistic regression, adjusting for age, sex, and factors associated with the dependent variable. In the logistic regression, the variables of hours of mobile phone use per day and hours spent on E-mail, SNS, online chat, internet, playing games, and viewing videos were each included as predictors. Coefficients of correlation between variables of time were not high (*r*: −0.025 to 0.569). *p*-values < 0.05 were considered statistically significant. All statistical analyses were performed using SPSS 20.0 (IBM Japan, Tokyo, Japan) for Windows.

## 3. Results

As shown in Table 1, the 295 students consisted of 173 (58.6%) boys and 122 (41.4%) girls, with the mean (standard deviation: *SD*) age of 16.2 (0.9) years (range: 15 to 19). Mobile phones were owned by 98.6% of participants (*n* = 291), and 92.9% (*n* = 274) owned a smartphone. Concerning hours of mobile phone use, 58.6% of participants used them for more than 2 h per day, and 10.5% used them for over 5 h per day.

Relationships between overall hours of mobile phone use per day and lifestyle, social support, insomnia, and depression were shown in Table 2. More hours per day of overall mobile phone use was associated with female sex (*p* < 0.001), non-participation in the school’s club activities (*p* < 0.001), late bedtime (*p* = 0.001), short hours of sleep (*p* = 0.006), and occasionally skipping breakfast (*p* = 0.007). Insomnia and depression were also associated with longer total hours of mobile phone use (*p* = 0.025 and *p* = 0.022, respectively).

Table 3 shows the associations between mobile phone use and insomnia or depression in multiple logistic regression analyses, adjusting for age, sex, and factors associated with insomnia or depression. Insomnia was more frequently found in students who used mobile phones for 5 h or more a day than those using mobile phones for less than 1 h (OR: 3.89, 95% confidence interval (CI): 1.21–12.49). Mobile phone use of 120 min or more for online chat was also associated with insomnia (OR: 2.81; 95% CI: 1.28–6.15), compared with mobile phone use of 30 min or less for online chat. Meanwhile, overall hours of mobile phone use was not related with depression. On the other hand, 120 min or more of mobile phone use for SNS (OR: 3.63; 95% CI: 1.20–10.98) and online chat (OR: 3.14; 95% CI: 1.42–6.95) was associated with depression, compared with mobile phone use of less than 30 min for SNS and online chat. Hours spent using a mobile phone for internet searching or video games was not linked with depression.

## 4. Discussion

The present study showed that excessively long hours of mobile phone use was associated with insomnia, particularly in students using mobile phones for 5 h or more per day compared with those using mobile phones for less than 1 h per day. On the other hand, no association was found between total hours of mobile phone use and depression. However, interestingly, long hours of mobile phone use for SNS or online chat were related to depression, particularly in students who spent 120 min or more on SNS and online chat, while hours spent using a mobile phone for internet searching, playing games or viewing videos was not associated with depression.

This study showed that long hours of mobile phone use was a risk factor for insomnia. It is also suggested that overuse of 5 h and more a day could be a marker of a higher risk of insomnia. To our knowledge, there are only two studies examining the association between sleep disturbances and hours of mobile phone use. Among adolescents in Hong Kong, long hours of mobile phone use were correlated with short sleep duration, poor sleep quality, and excessive daytime sleepiness [20]. Another study in Japanese high school students reported that long hours of mobile phone use was associated with short sleep time and fatigue [23]. Both findings support an association between long hours of mobile phone use and sleep disturbances, as shown in this study. In this study, among adolescents using mobile phones for 5 h or more a day, 61.3% reported a bedtime after 00:00, and 67.8% had a sleeping time of less than 6 h per day. Thus, mobile phone overuse was linked to disturbances in sleep habits, which is known to be a risk factor for insomnia [37]. It is, therefore, considered that mobile phone overuse can cause impaired sleep habits, thus contributing to insomnia.

In the present study, depression was not associated with total hours of mobile phone use. However, long hours spent using mobile phones for SNS and online chat was related to depression, while hours spent using mobile phones for internet searching, playing games or viewing videos was not linked with depression. The SNS (e.g., Facebook, Twitter or Instagram) and online chat (e.g., Line, Skype, Kakao Talk) are popular online communication tools among adolescents [38]. Some earlier studies have indicated that their use is associated with mental health problems [39,40,41,42]. Additionally, it is reported that internet addiction can be predicted by the use of SNS and chat rooms [39,40]; and that the use of SNS contributed to psychological distress, suicidal ideation and attempts [41,42]. Sampasa-Kanyinga and Lewis [42] reported that using SNS for more than 2 h every day was independently associated with poor self-rating of mental health, high levels of psychological distress and suicidal ideation. The present study confirmed that 2 h and more a day spent using mobile phones for SNS and online chat could increase risks of depression. SNS and online chat enable one to communicate and interact with a large number of people. Hence, young users may spend more time on them [43]. However, the overuse of SNS and online chat sometimes undermines well-being and life satisfaction [44], increases risk of cyberbullying victimization [41], and can also relate to depression in adolescents.

The present study showed that female adolescents used mobile phones for longer hours per day than males. Previous studies also indicated that women tended to overuse online applications for social function or communication, such as e-mail, chat, and SNS [45,46,47]. Moreover, overuse of online communication was more likely to cause sleep disturbances and stress among women [48]. Thus, female adolescents in particular should be careful to prevent mobile phone overuse.

The Japanese government has concerns about various problems such as bullying, crime or addiction among adolescents through the use of the internet, and insists on educating school students about appropriate mobile phone use, including the restriction on hours of mobile phone use [49]. The present findings may suggest that appropriate use of mobile phones in adolescents needs to be considered.

The present study had some limitations. The present subjects were limited to participants in a single high school in central Japan, so the findings from this study cannot be largely generalized to other areas and countries. Another limitation was that the information on hours of mobile phone use was not very precise because the information was obtained using a self-administered questionnaire. Therefore, “5 h and more” is a rough criterion for overuse. Finally, this was a cross-sectional study. Hence, the present findings do not show a causal relation. Even so, this study showed meaningful findings. Mobile phone overuse could be linked to impaired sleep habits, and consequently to insomnia. It was suggested that 5 h and more of phone use in particular could increase risks of insomnia. Additionally, the overuse of mobile phones—120 min or more—for SNS and online chat might be related to depression among adolescents. Appropriate use of mobile phones should be considered.

## 5. Conclusions

The present study found that long hours of mobile phone use was associated with insomnia, particularly in students using mobile phones for 5 h or more a day. Additionally, long hours spent using mobile phones for SNS or online chat was related to depression, particularly in students who spent 2 h or more on SNS and online chat. Appropriate use of mobile phones should be considered in order to prevent sleep disturbances and the impairment of mental health among adolescents.

## Figures and Tables

**Table 1:** Characteristics of students.

**Parameters**	**Mean**	**SD**
Age	16.2	0.9
Athens Insomnia Scale (range 0–20)	4.1	3.2
CES-D (range 2–47)	16.2	8.5
**Parameters**	***n***	**%**
School grade		
10th	102	35.1
11th	96	33.0
12th	93	32.0
Sex		
Men	173	58.6
Women	122	41.4
Own a mobile phone		
smartphone	274	92.9
the conventional phone	17	5.8
None	4	1.4
Total hours of mobile phone use (h/day)		
None	8	2.7
<1 h	32	10.8
1 to <2 h	82	27.8
2 to <3 h	80	27.1
3 to <4 h	38	12.9
4 to <5 h	24	8.1
≥5 h	31	10.5
E-mail (min/day)		
None	144	49.1
<30 min	85	29.0
30 to <60 min	33	11.3
60 to <120 min	16	5.5
≥120 min	15	5.1
SNS (minutes/day)		
None	114	38.8
<30 min	72	24.5
30 to <60 min	48	16.3
60 to <120 min	40	13.6
≥120 min	20	6.8
Online chat (min/day)		
None	18	6.1
<30 min	77	26.1
30 to <60 min	67	22.7
60 to <120 min	68	23.1
≥120 min	65	22.0
Internet search (min/day)		
None	33	11.2
<30 min	148	50.3
30 to <60 min	83	28.2
60 to <120 min	20	6.8
≥120 min	10	3.4
Play games (min/day)		
None	53	18.0
<30 min	75	25.4
30 to <60 min	98	33.2
60 to <120 min	49	16.6
≥120 min	20	6.8
Watch videos (min/day)		
None	40	13.6
<30 min	97	32.9
30 to <60 min	81	27.5
60 to <120 min	38	12.9
≥120 min	39	13.2
Insomnia		
no problem	215	72.9
insomnia	80	27.1
Depression		
no problem	182	61.7
depression	113	38.3

Data are expressed as mean (SD) and frequency (%). SD: Standard deviation; CES-D: Center for Epidemiological Studies-Depression scale; SNS: Social networking sites.

**Table 2:** The relationship between total hours of mobile phone use (h/day) and lifestyle, social support, insomnia, and depression.

Variables	<1 h		1 to <3 h		3 to <5 h		≥5 h		*p*
	Mean	SD	Mean	SD	Mean	SD	Mean	SD	
Age	16.3	1.1	16.1	0.9	16.3	1.0	16.3	0.7	0.486
Athens Insomnia Scale	3.6	3.9	4.0	3.2	3.9	2.3	5.2	3.3	0.050
CES-D	14.0	7.2	16.0	8.5	15.8	7.9	21.2	9.6	0.013
Social support	5.6	1.3	5.4	1.1	5.6	1.1	5.6	1.3	0.731
	***n***	**%**	***n***	**%**	***n***	**%**	***n***	**%**	***p***
Insomnia									0.025
no problem	32	80.0	119	73.5	49	79.0	15	48.4	
insomnia	8	20.0	43	26.5	13	21.0	16	51.6	
Depression									0.022
no problem	27	67.5	105	64.8	37	59.7	13	41.9	
depression	13	32.5	57	35.2	25	40.3	18	58.1	
Sex									<0.001
Men	32	80.0	102	63.0	31	50.0	8	25.8	
women	8	20.0	60	37.0	31	50.0	23	74.2	
Participation in school club activities									<0.001
sports club	21	52.5	75	46.6	21	33.9	6	19.4	
culture club	15	37.5	57	35.4	25	40.3	13	41.9	
None	4	10.0	29	18.0	16	25.8	12	38.7	
Bedtime									0.001
earlier than 23:00	10	25.0	30	18.5	4	6.5	3	9.7	
at 23:00–00:00	17	42.5	75	46.3	29	46.8	9	29.0	
at 00:00–01:00	9	22.5	38	23.5	24	38.7	10	32.3	
later than 01:00	4	10.0	19	11.7	5	8.1	9	29.0	
Wake-up time									0.719
earlier than 06:00	10	25.0	43	26.7	15	24.2	8	25.8	
at 06:00-07:00	23	57.5	80	49.7	32	51.6	16	51.6	
later than 07:00	7	17.5	38	23.6	15	24.2	7	22.6	
Hours spent sleeping									0.006
<5 h	3	7.7	15	9.3	4	6.6	7	22.6	
5 to <6 h	12	30.8	37	23.0	20	32.8	14	45.2	
6 to <7 h	16	41.0	64	39.8	26	42.6	7	22.6	
≥7 h	8	20.5	45	28.0	11	18.0	3	9.7	
Eating breakfast									0.007
eat daily	36	90.0	135	83.9	53	85.5	19	61.3	
occasionally do not eat	4	10.0	26	16.1	9	14.5	12	38.7	
Talking with family									0.206
talk daily	32	80.0	140	86.4	57	91.9	27	87.1	
occasionally do not talk	8	20.0	22	13.6	5	8.1	4	12.9	

Data are expressed as mean (SD) and frequency (%); *p* values by Kruskal–Wallis test and Mantel–Haenszel test for trend; CES-D is the Center for Epidemiological Studies-Depression scale.

**Table 3:** The associations between mobile phone use and insomnia or depression using multiple logistic regression analyses.

Variables	Insomnia				Depression			
	Crude OR		Adjusted OR ^†^		Crude OR		Adjusted OR ^‡^	
	OR (95% CI)	*p*	OR (95% CI)	*p*	OR (95% CI)	*p*	OR (95% CI)	*p*
Total hours of mobile phone use (h/day)								
<1 h	reference		reference		reference		reference	
1 to 3 h	1.45 (0.62–3.38)	0.395	1.30 (0.52–3.28)	0.575	1.13 (0.54–2.35)	0.749	0.98 (0.41–2.34)	0.961
3 to 5 h	1.06 (0.40–2.85)	0.906	1.00 (0.34–2.93)	0.996	1.40 (0.61–3.23)	0.426	1.26 (0.47–3.37)	0.645
≥5 h	4.27 (1.50–12.16)	0.007	3.89 (1.21–12.49)	0.023	2.88 (1.09–7.61)	0.033	2.21 (0.69–7.06)	0.181
E-mail (min/day)								
<30 min	reference		reference		reference		reference	
30 to 60 min	1.17 (0.53–2.60)	0.698	1.06 (0.44–2.57)	0.891	1.36 (0.65–2.84)	0.412	1.57 (0.65–3.78)	0.313
60 to 120 min	0.62 (0.17–2.26)	0.470	0.66 (0.17–2.59)	0.547	0.74 (0.25–2.21)	0.591	0.51 (0.13–2.00)	0.331
≥120 min	1.35 (0.44–4.10)	0.600	1.66 (0.50–5.56)	0.412	0.82 (0.27–2.47)	0.719	0.75 (0.21–2.73)	0.664
SNS (min/day)								
<30 min	reference		reference		reference		reference	
30 to 60 min	1.31 (0.64–2.71)	0.461	1.67 (0.75–3.70)	0.210	1.17 (0.61–2.26)	0.638	1.38 (0.62–3.06)	0.435
60 to 120 min	2.61 (1.28–5.35)	0.009	3.18 (1.40–7.22)	0.006	1.77 (0.89–3.53)	0.107	1.90 (0.83–4.35)	0.128
≥120 min	2.36 (0.90–6.15)	0.080	2.57 (0.89–7.43)	0.082	2.93 (1.14–7.53)	0.026	3.63 (1.20–10.98)	0.023
Online chat (minutes/day)								
<30 min	reference		reference		reference		reference	
30 to 60 min	1.11 (0.53–2.32)	0.791	1.10 (0.49–2.44)	0.824	1.54 (0.80–2.96)	0.199	1.96 (0.92–4.18)	0.082
60 to 120 min	1.18 (0.57–2.44)	0.667	1.52 (0.69–3.38)	0.302	1.16 (0.60–2.26)	0.656	1.56 (0.71–3.44)	0.272
≥120 min	2.35 (1.17–4.70)	0.016	2.81 (1.28–6.15)	0.010	2.50 (1.30–4.80)	0.006	3.14 (1.42–6.95)	0.005
Internet search (minutes/day)								
<30 min	reference		reference				reference	
30 to 60 min	1.48 (0.82–2.68)	0.194	1.44 (0.76–2.74)	0.262	1.40 (0.82–2.39)	0.219	1.17 (0.62–2.20)	0.628
60 to 120 min	6.76 (2.53–18.1)	<0.001	4.83 (1.68–13.88)	0.003	3.75 (1.42–9.88)	0.008	1.38 (0.45–4.24)	0.573
≥120 min	2.43 (0.65–9.03)	0.186	3.04 (0.74–12.46)	0.123	3.03 (0.82–11.13)	0.096	3.89 (0.91–16.64)	0.067
Playing games (minutes/day)								
<30 min	reference		reference		reference		reference	
30 to 60 min	1.19 (0.65–2.17)	0.570	1.32 (0.69–2.52)	0.398	0.95 (0.55–1.64)	0.846	0.98 (0.52–1.85)	0.957
60 to 120 min	1.52 (0.74–3.12)	0.257	1.52 (0.69–3.36)	0.298	1.71 (0.88–3.33)	0.114	1.50 (0.68–3.30)	0.310
≥120 min	1.34 (0.48–3.79)	0.580	1.58 (0.52–4.80)	0.417	1.46 (0.56–3.78)	0.437	1.38 (0.46–4.12)	0.563
Viewing of videos (minutes/day)								
<30 min	reference		reference		reference		reference	
30 to 60 min	0.83 (0.44–1.59)	0.580	0.77 (0.38–1.56)	0.475	1.03 (0.58–1.84)	0.909	1.08 (0.55–2.09)	0.831
60 to 120 min	1.70 (0.79–3.65)	0.173	1.56 (0.70–3.50)	0.277	1.67 (0.81–3.45)	0.167	1.57 (0.69–3.57)	0.281
≥120 min	1.46 (0.68–3.14)	0.337	1.54 (0.68–3.47)	0.298	1.59 (0.77–3.27)	0.208	1.47 (0.65–3.31)	0.352

Data are expressed as odds ratio (OR) and 95% confidence interval (CI) of logistic regression analysis. *p* value by multiple logistic regression analysis; ^†^ adjusting for age, sex, talking with family, and social support; ^‡^ adjusting for age, sex, talking with family, social support, and hours spent sleeping.
